# Supplementary material for: Determinants of Quality of Life in Adult Patients with Chronic Non-Bacterial Osteomyelitis (CNO) of the Sternocostoclavicular Region (SCCH): A Dutch Single Center Study
Source: J Clin Med. 2022 Mar 27;11(7):1852. doi: 10.3390/jcm11071852 (PMC9000162; doi:10.3390/jcm11071852)
Supplement: Supplementary file 1 [file jcm-11-01852-s001.zip › jcm-1634994-supplementary.pdf]

Journal of Clinical Medicine

SUPPLEMENTARY MATERIALS

Table S1. Correlations between quality of life (Short Form-36) and clinical symptoms (Brief Pain inventory/ Shoulder Rating Questionnaire) in CNO/SCCH patients.

BPI/SRQ domain	Pain Severity		Pain Interference		Worst pain 24h		Average pain		Pain at BPI completion		Impairment in shoulder function (daily activities)		Impairment in shoulder function (sports/recreation)	
**SF-36 domain**	***R***	**Sig.**	***R***	**Sig.**	***R***	**Sig.**	***R***	**Sig.**	***R***	**Sig.**	***R***	**Sig.**	***R***	**Sig.**
Physical function	−0.63	**0.000**	−0.71	**0.000**	−0.65	**0.000**	−0.65	**0.000**	−0.62	**0.001**	0.615	**0.000**	0.547	**0.000**
Role physical function	−0.55	**0.000**	−0.65	**0.000**	−0.61	**0.000**	−0.55	**0.000**	−0.56	**0.000**	0.595	**0.000**	0.469	**0.000**
Bodily pain	−0.28	0.012	−0.16	0.143	−0.34	**0.002**	−0.29	**0.008**	−0.27	0.016	0.173	0.128	0.081	0.476
General health	−0.44	**0.000**	−0.46	**0.000**	−0.41	**0.000**	−0.42	**0.000**	−0.43	**0.000**	0.223	0.048	0.227	0.043
Vitality	−0.51	**0.000**	−0.63	**0.000**	−0.43	**0.000**	−0.51	**0.000**	−0.51	**0.000**	0.452	**0.000**	0.390	**0.000**
Social function	−0.43	**0.000**	−0.47	**0.000**	−0.46	**0.000**	−0.44	**0.000**	−0.43	0.017	0.323	**0.004**	0.274	**0.010**
Role emotional	−0.23	0.041	−0.34	**0.002**	−0.25	0.025	−0.23	0.040	−0.23	0.036	0.261	0.021	0.168	0.139
Mental health	−0.36	**0.001**	−0.58	**0.000**	−0.28	**0.01**	−0.36	**0.001**	−0.33	**0.003**	0.346	**0.002**	0.301	**0.007**

Level of significance set at $p \leq 0.01$ are given in bold.

Table S2. Correlations between Short Form-36 and Brief-Illness Perception Questionnaire domains in CNO/SCCH patients.

B-IPQ domain	Consequences		Timeline		Personal Control		Treatment control		Identity		Illness concern		Coherence		Emotional response	
**SF-36 domain**	***R***	**Sig.**	***R***	**Sig.**	***R***	**Sig.**	***R***	**Sig.**	***R***	**Sig.**	***R***	**Sig.**	***R***	**Sig.**	***R***	**Sig.**
Physical function	-0.61	**0.000**	-0.18	0.116	0.17	0.130	0.05	0.669	−0.51	**0.000**	−0.32	**0.004**	−0.14	0.216	−0.37	**0.001**
Role physical function	-0.70	**0.000**	-0.15	0.176	0.26	0.022	0.13	0.256	−0.65	**0.000**	−0.46	**0.001**	−0.06	0.633	−0.48	**0.000**
Bodily pain	0.18	0.103	0.05	0.686	0.24	0.036	0.20	0.082	−0.21	0.058	−0.17	0.122	0.003	0.976	−0.10	0.361
General health	-0.46	**0.000**	-0.30	0.008	0.12	0.311	0.02	0.891	−0.43	**0.001**	−0.39	**0.000**	-0.06	0.625	−0.37	**0.001**
Vitality	-0.50	**0.000**	-0.11	0.353	0.19	0.089	0.17	0.128	−0.28	0.012	−0.56	**0.000**	0.142	0.213	−0.72	**0.000**
Social function	-0.40	**0.000**	-0.03	0.818	0.21	0.063	0.10	0.410	−0.30	**0.007**	−0.39	**0.000**	0.06	0.586	−0.33	**0.003**
Role emotional	-0.37	**0.001**	-0.01	0.915	0.16	0.156	0.03	0.801	−0.18	0.112	−0.42	**0.000**	0.19	0.105	−0.63	**0.000**
Mental health	-0.40	**0.000**	-0.15	0.171	0.29	**0.009**	0.05	0.664	−0.18	0.114	−0.51	**0.000**	0.22	0.056	−0.75	**0.000**

Level of significance set at $p \leq 0.01$ are given in bold.

Table S3. Correlations between Utrecht Coping List domains and Short Form-36 domains in CNO/SCCH patients.

**SF-36 domain**	**Physical function**		**Social function**		**Role physical function**		**Role emotional**		**Mental health**		**Vitality**		**Bodily pain**		**General health**	
**UCL domain**	**R**	**Sig.**	**R**	**Sig.**	**R**	**Sig.**	**R**	**Sig.**	**R**	**Sig.**	**R**	**Sig.**	**R**	**Sig.**	**R**	**Sig.**
Active coping	-0.146	0.195	-0.139	0.217	-0.101	0.371	-0.046	0.686	-0.045	0.691	-0.104	0.358	0.004	0.971	**-0.316**	**0.004**
Palliative coping	0.031	0.784	0.001	0.992	0.034	0.763	-0.004	0.975	0.251	0.025	0.205	0.068	0.035	0.760	-0.085	0.451
Avoiding coping	0.090	0.432	-0.250	0.027	-0.110	0.338	-0.203	0.076	-0.139	0.226	-0.124	0.278	-0.118	0.302	-0.027	0.813
Social support	0.102	0.373	0.179	0.116	0.161	0.158	0.166	0.150	0.211	0.064	0.139	0.224	0.108	0.348	0.114	0.320
Passive coping	0.099	0.387	-0.013	0.909	-0.024	0.833	0.018	0.873	0.052	0.649	0.045	0.693	-0.186	0.101	-0.025	0.826
Express emotions	0.050	0.659	0.004	0.975	0.038	0.740	0.048	0.673	0.036	0.752	0.069	0.544	-0.087	0.442	-0.025	0.828
Reassuring thoughts	0.025	0.825	0.002	0.986	0.001	0.990	-0.201	0.074	0.064	0.567	-0.004	0.974	0.103	0.360	-0.012	0.916

Level of significance set at $p \leq 0.01$ are given in bold.
